# SPINDLY mediates *O*-fucosylation of hundreds of proteins and sugar-dependent growth in Arabidopsis

**DOI:** 10.1093/plcell/koad023

**Published:** 2023-02-06

**Authors:** Yang Bi, Ruben Shrestha, Zhenzhen Zhang, Chuan-Chih Hsu, Andres V Reyes, Sumudu Karunadasa, Peter R Baker, Jason C Maynard, Yang Liu, Amirmansoor Hakimi, Daniel Lopez-Ferrer, Tahmid Hassan, Robert J Chalkley, Shou-Ling Xu, Zhi-Yong Wang

**Affiliations:** Department of Plant Biology, Carnegie Institution for Science, Stanford, California 94305, USA; Department of Plant Biology, Carnegie Institution for Science, Stanford, California 94305, USA; Department of Plant Biology, Carnegie Institution for Science, Stanford, California 94305, USA; Department of Plant Biology, Carnegie Institution for Science, Stanford, California 94305, USA; Institute of Plant and Microbial Biology, Academia Sinica, Taipei 115, Taiwan; Department of Plant Biology, Carnegie Institution for Science, Stanford, California 94305, USA; Carnegie Mass Spectrometry Facility, Carnegie Institution for Science, Stanford, California 94305, USA; Department of Plant Biology, Carnegie Institution for Science, Stanford, California 94305, USA; Department of Pharmaceutical Chemistry, University of California at San Francisco, San Francisco, California 94143, USA; Department of Pharmaceutical Chemistry, University of California at San Francisco, San Francisco, California 94143, USA; ThermoFisher Scientific, San Jose, California 95134, USA; ThermoFisher Scientific, San Jose, California 95134, USA; ThermoFisher Scientific, San Jose, California 95134, USA; ThermoFisher Scientific, Somerset, New Jersey 08873, USA; Department of Pharmaceutical Chemistry, University of California at San Francisco, San Francisco, California 94143, USA; Department of Plant Biology, Carnegie Institution for Science, Stanford, California 94305, USA; Carnegie Mass Spectrometry Facility, Carnegie Institution for Science, Stanford, California 94305, USA; Department of Plant Biology, Carnegie Institution for Science, Stanford, California 94305, USA

## Abstract

The recent discovery of SPINDLY (SPY)-catalyzed protein *O*-fucosylation revealed a novel mechanism for regulating nucleocytoplasmic protein functions in plants. Genetic evidence indicates the important roles of SPY in diverse developmental and physiological processes. However, the upstream signal controlling SPY activity and the downstream substrate proteins *O*-fucosylated by SPY remain largely unknown. Here, we demonstrated that SPY mediates sugar-dependent growth in Arabidopsis (*Arabidopsis thaliana*). We further identified hundreds of *O*-fucosylated proteins using lectin affinity chromatography followed by mass spectrometry. All the *O*-fucosylation events quantified in our proteomic analyses were undetectable or dramatically decreased in the *spy* mutants, and thus likely catalyzed by SPY. The *O*-fucosylome includes mostly nuclear and cytosolic proteins. Many *O*-fucosylated proteins function in essential cellular processes, phytohormone signaling, and developmental programs, consistent with the genetic functions of SPY. The *O*-fucosylome also includes many proteins modified by *O*-linked *N*-acetylglucosamine (*O*-GlcNAc) and by phosphorylation downstream of the target of rapamycin (TOR) kinase, revealing the convergence of these nutrient signaling pathways on key regulatory functions such as post-transcriptional/translational regulation and phytohormone responses. Our study identified numerous targets of SPY/*O*-fucosylation and potential nodes of crosstalk among sugar/nutrient signaling pathways, enabling future dissection of the signaling network that mediates sugar regulation of plant growth and development.

## Teaser

A proteomic profiling of *O*-fucose-modified intracellular proteins in Arabidopsis reveals mechanisms of sugar-dependent growth regulation and crosstalk among nutrient-sensing pathways.

## Introduction

Nutrient sensing and signaling are critical for homeostasis, growth, and development in all organisms. Extensive studies in animals have established that posttranslational modification (PTM) of nucleocytoplasmic proteins by *O*-linked *N*-acetylglucosamine (*O*-GlcNAc), catalyzed by *O*-GlcNAc transferase (OGT), is an essential nutrient-sensing mechanism that regulates protein functions and cellular homeostasis according to nutrient and energy status (Yang and Qian 2017; Ong et al. 2018; Ma et al. 2021). Animals have only one OGT, but *Arabidopsis thaliana* has two OGT homologs named SPINDLY (SPY) and SECRET AGENT (SEC). SPY and SEC represent two distinct evolutionary clades present in all plants and red algae, whereas the animal and fungi kingdoms contain only homologs of SEC but not SPY (Olszewski et al. 2010; Sun 2021). While SEC appears to be a canonical *O*-GlcNAc transferase, SPY was recently shown to be a protein *O*-fucose transferase (POFUT) (Zentella et al. 2016, 2017; Sun 2021). This study uncovers protein *O*-fucosylation as a novel signaling mechanism and, together with genetic studies, suggests that SPY regulates diverse developmental processes through *O*-fucosylation of many nucleocytoplasmic proteins that have yet to be identified.

Genetic studies have indicated that SPY and SEC play redundant essential roles in plant viability and have complex interactions in various signaling and developmental pathways (Hartweck et al. 2002). The *spy* single mutants display a range of developmental defects including slim and small seedlings, pale leaves, early flowering, reduced fertility, and constitutive responses to gibberellic acid (GA) (Jacobsen and Olszewski 1993). Further detailed analyses have provided evidence for prominent functions of SPY in diverse processes, which include GA and cytokinin signaling, circadian clock, flowering, root development, light responses, abscisic acid (ABA) responses, abiotic stresses, and pathogen responses (Tseng et al. 2004; Qin et al. 2011; Steiner et al. 2012; Cui et al. 2014; Zentella et al. 2017; Zhang et al. 2019; Mutanwad et al. 2020; Wang et al. 2020; Sun 2021). On the other hand, the *sec* single mutants display only subtle phenotypes such as short hypocotyls and early flowering (Zentella et al. 2016). The *spy sec* double mutant, however, is embryo lethal (Hartweck et al. 2002), suggesting additive effects on certain cellular functions. These observations suggest that SPY and SEC play redundant essential roles in growth and viability, while SPY plays prominent roles in broad developmental and physiological processes.

The functions of *O*-fucosylation have been characterized for only a handful of proteins in plants. The first identified substrates of SPY-mediated *O*-fucosylation are the DELLA proteins, repressors of GA signaling and plant growth (Zentella et al. 2017). DELLAs are known substrates of SEC-mediated *O*-GlcNAcylation, but no OGT activity has been detected for SPY (Zentella et al. 2016). Instead, SPY was recently found to catalyze *O*-fucosylation of DELLA on serine and threonine residues using GDP-fucose as a donor substrate (Zentella et al. 2017). *O*-fucosylation activates and *O*-GlcNAcylation represses DELLA function by increasing and decreasing DELLA's interactions with its target transcription factors, respectively (Zentella et al. 2016, 2017). The antagonistic effects of *O*-GlcNAc and *O*-fucose modifications on DELLA and the lethal phenotype of the *spy sec* double mutant suggest that SPY and SEC can have additive or antagonistic effects on different proteins.

In addition to DELLAs, PSEUDO RESPONSE REGULATOR 5 (PRR5), a circadian clock component, has been shown to be destabilized by SPY-mediated *O*-fucosylation, but is not modified by *O*-GlcNAcylation (Wang et al. 2020). More recently, a transcriptional and RNA splicing regulator, named AtACINUS, was reported to be modified by *O*-fucosylation and *O*-GlcNAcylation (Bi et al. 2021). The human homolog acinus (apoptotic chromatin condensation inducer in the nucleus) is known as a hub in the protein interaction network that regulates gene expression (Murachelli et al. 2012). Similarly, AtACINUS interacts with chromatin remodeling factors and RNA splicing factors. Several AtACINUS-dependent intron splicing events were altered in the *spy* and *sec* mutants, providing evidence for functions of *O*-fucose and *O*-GlcNAc in regulating AtACINUS functions and alternative RNA splicing (Bi et al. 2021). SPY also interacts with bHLH transcription factors TCP14 and TCP15 to promote cytokinin responses (Steiner et al. 2012, 2016). TCP14 and TCP15 are *O*-GlcNAcylated by SEC, but it is unclear how *O*-GlcNAcylation affects their functions and whether they are also *O*-fucosylated (Steiner et al. 2012, 2016). These genetic and molecular studies have indicated that SPY-catalyzed protein *O*-fucosylation is an important cellular signaling mechanism that regulates diverse developmental processes and shares essential functions with SEC/*O*-GlcNAcylation.

Studies in animals established *O*-GlcNAcylation of nucleocytoplasmic proteins as a nutrient-sensing mechanism that monitors diverse metabolic pathways. Nevertheless, the signals that control and are transduced by SPY and SEC remain unknown in plants. Proteomic studies have identified hundreds of *O*-GlcNAcylated proteins (substrates of SEC), which mostly play important regulatory roles (Xu et al. 2017). Identification of the substrate proteins *O*-fucosylated by SPY is required to advance our understanding of the global functions of SPY/*O*-fucosylation and its interplay with SEC/*O*-GlcNAcylation (Sun 2021).

In this study, we demonstrate SPY's function in sugar signaling and identify targets of SPY-mediated *O*-fucosylation. We developed an affinity chromatography method using *Aleuria aurantia* lectin (AAL) to identify *O*-fucosylated peptides in Arabidopsis. Using this method, we identified 943 *O*-fucosylated peptides in 467 proteins, generating an *O*-fucosylation profile in plants. Quantitative proteomic comparison between wild-type (WT) and *spy* mutants demonstrated an essential role for SPY in protein *O*-fucosylation. Our proteomic study provides molecular evidence revealing the broad regulatory functions of *O*-fucosylation in key cellular and developmental processes, as well as in all major phytohormone signaling pathways. Comparing the targets of the SPY, SEC, and TOR pathways provides a system-level view of network organization and reveals potential junctions of crosstalk and overlapping actions among these nutrient-sensing pathways in regulating different cellular, developmental, and physiological processes. Our study reveals numerous target proteins and the network framework for future dissection and engineering of the molecular networks that couple nutrient and energy status with growth regulation in plants.

## Results

### SPY plays an essential role in sugar-dependent plant growth

**Figure 1.**
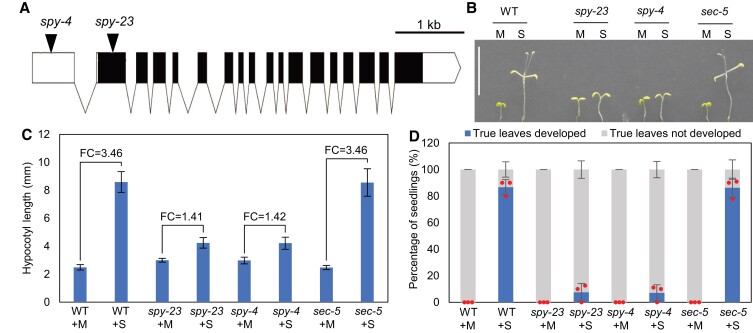
SPY plays an essential role in sugar-dependent protein *O*-fucosylation and plant growth. A) A schematic diagram showing the localizations of *spy-4* and *spy-23* T-DNA insertion sites in the *SPY* gene. Untranslated regions (UTRs), exons, and introns are represented by empty rectangles, filled rectangles, and zigzag lines, respectively. B–D) WT, *spy-23, spy-4,* and *sec-5* seedlings were grown on ½-MS supplemented with 1% sucrose (S) or with mannitol as a control (M) for 4 d under light and then transferred to dark for 8 d. Representative seedlings are displayed in (B). Scale bar is 10 mm (B). The measurements of hypocotyl lengths and fold changes (FC, +S/+M) are shown in (C). The bar graph shows mean ± Sem (*n* = 10). ANOVA analysis shows that sucrose-induced hypocotyl elongation of *spy-23* and *spy-4* is significantly different from that of the WT (*P*-values < 2e-16). The percentages of seedlings that developed obvious true leaves are shown in (D). The bar graphs show mean ± Sem of the averages of three replicate experiments each including at least 8 seedlings. ANOVA analysis shows that sucrose-induced true leaves development of *spy-23* and *spy-4* is significantly different from that of the WT (*P*-values = 2.82e-7 and 1.93e-7, respectively).

The *spy* and *sec* mutants have been characterized extensively in the context of a wide range of developmental and physiological processes. However, it has remained unclear whether their phenotypes are related to nutrient sensing. To test the function of SPY in sugar-dependent growth, we grew Arabidopsis wild-type (WT), as well as *sec5* (Xing et al. 2018), *spy-4* (Swain et al. 2001) and our newly identified *spy-23* T-DNA insertion mutants on media containing 1% sucrose or, as a control, mannitol, which is a sugar alcohol that cannot be metabolized by plants. The expression levels of SEC (Xing et al. 2018) and SPY (Swain et al. 2001) were reported to be severely decreased in these mutants. After 4 d of growth under light, the seedlings were put in the dark to deplete the sugar supply from photosynthesis (Fig. 1, A–D). All seedlings on mannitol media stopped growing, while the WT and *sec* seedlings on sucrose-supplemented media continued to grow. In contrast, the *spy* mutants on sucrose-supplemented media grew very little compared with WT and *sec*, indicating that SPY, but not SEC, is required for sugar-dependent growth.

### AAL-based chromatography effectively identifies *O*-fucosylated proteins as potential SPY substrates in *Arabidopsis*

**Figure 2.**
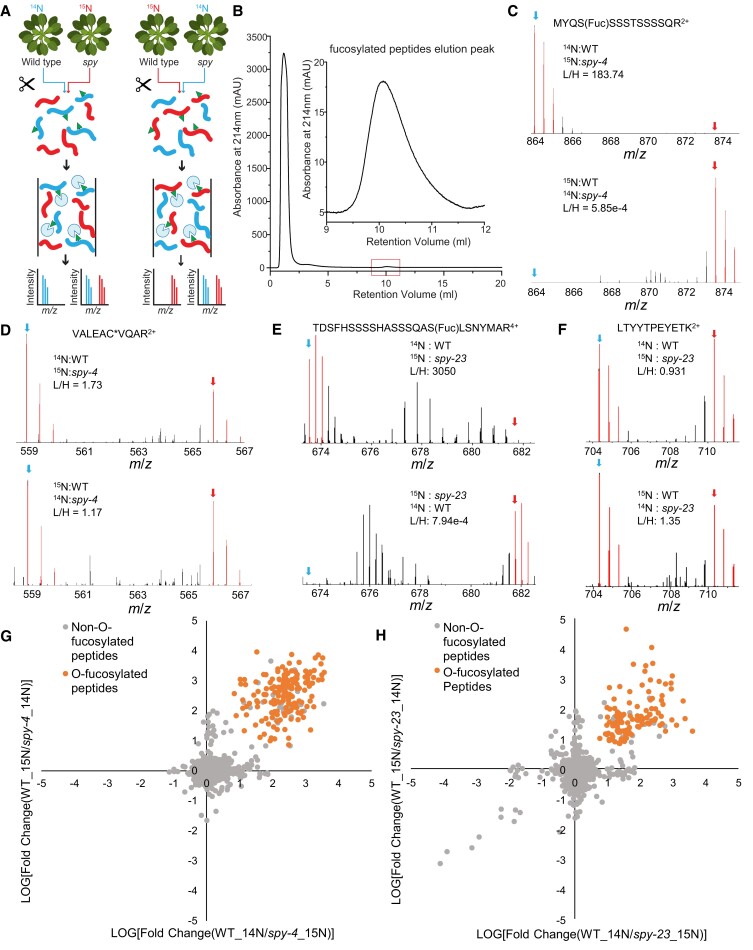
AAL-based chromatography effectively identifies *O*-fucosylated proteins as SPY substrates in *Arabidopsis*. A) Workflow for stable isotope-labeling, AAL-enrichment, and mass spectrometry. Total proteins from ^14^N- and ^15^N-labeled WT and *spy* mutants are extracted, mixed, and trypsin digested. *O*-fucosylated peptides (shown with triangle tag) are captured using AAL-agarose beads and analyzed by LC-MS/MS. B) The chromatogram of absorbance at 214 nm of AAL-chromatography shows enrichment of *O*-fucose-modified peptides after elution with *O*-fucose (the inset shows an amplified view of the elution peak). C–F) Selected MS1 spectra show the relative abundance of *O*-fucosylated (C and E) and non-*O*-fucosylated (D and F) peptides in WT compared with *spy*. The peptides are from ABA-RESPONSIVE KINASE SUBSTRATE 2 (AKS2, AT1G05805.1) (C), RIBULOSE-BISPHOSPHATE CARBOXYLASES (RBCL, ATCG00490.1) (D, F), and WITH NO LYSINE (K) KINASE 4 (WNK4, AT5G58350.1) (E). C*) Carbamidomethylated cysteine. G, H) Scatter plots of log_10_ ratios of WT/*spy-4* (G) and WT/*spy-23* (H) for peptides (data for *O*-fucosylated peptides are in orange color) that were detected and quantified in both isotope-switched replicate experiments.

To identify *O*-fucosylated proteins from the *Arabidopsis* proteome, we developed an affinity chromatography method using AAL (Bandini et al. 2016) to enrich *O*-fucosylated peptides for analysis with mass spectrometry (MS). To determine whether the identified peptides were modified by SPY-mediated *O*-fucosylation, we used stable isotope labeling mass spectrometry (SIL-MS) to quantitatively compare peptide abundance between WT and the *spy* mutants (Fig. 2A). WT and *spy-23* or *spy-4* seedlings were grown on media containing the ^14^N or ^15^N stable isotope, with the isotopes reversed in repeat experiments (Fig. 2A; Supplemental Fig. S1). The ^14^N- or ^15^N-labeled tissues (WT and *spy-4*) or proteins (WT and *spy-23*) were mixed (^14^N-labeled WT and ^15^N-labeled *spy* were mixed in one experiment and those of ^14^N-labeled *spy* and ^15^N-labeled WT were mixed in the replicate experiment). Total protein was digested with trypsin. The peptides were purified by chromatography using a column packed with AAL-agarose beads. The *O*-fucosylated peptides were eluted with 10 mM L-fucose solution (Fig. 2B). The peptides were analyzed by liquid chromatography-tandem mass spectrometry (LC-MS/MS) using either high-resolution and high-accuracy Orbitrap Q-Exactive HF or Eclipse mass spectrometer with electron-transfer dissociation (ETD) (see Materials and Methods for details).

The MS/MS spectra identified 170 *O*-fucosylated peptides in WT and two in *spy-4* among a total of 3142 peptides whose identification was supported by both ^14^N- and ^15^N- labeled peptides (Supplemental Fig. S1, Supplemental Datasets S1 and S2), and 116 *O*-fucosylated peptides in WT and 40 in *spy-23* among a total of 4458 peptides (Supplemental Fig. S1, Supplemental Datasets S3 and S4) using the same criteria. Examination of MS1 spectra indicated that the *O*-fucosylated peptide peaks were near noise level in the *spy* mutants (Fig. 2, C–F). Quantitation of the intensity of isotopic peaks showed that all 170 *O*-fucosylated peptides were over six-fold more abundant in WT than in *spy-4*, whereas only 30 (0.95%) of the unmodified peptides showed similar differential abundance in WT and *spy-4* (Fig. 2G; Supplemental Datasets S1 and S2). Similarly, all 116 *O*-fucosylated peptides were over five-fold more abundant in WT than in *spy-23*, whereas only 17 (0.38%) of the unmodified peptides showed similar differences (Fig. 2H; Supplemental Datasets S3 and S4). The two datasets overlapped by 75 peptides and together show SPY-dependent *O*-fucosylation of 211 peptides from 150 proteins. These results indicate that our AAL-affinity chromatography coupled with the mass spectrometry method can specifically identify *O*-fucosylated proteins that are SPY substrates.

### Hundreds of nucleocytoplasmic proteins are *O*-fucosylated

**Figure 3.**
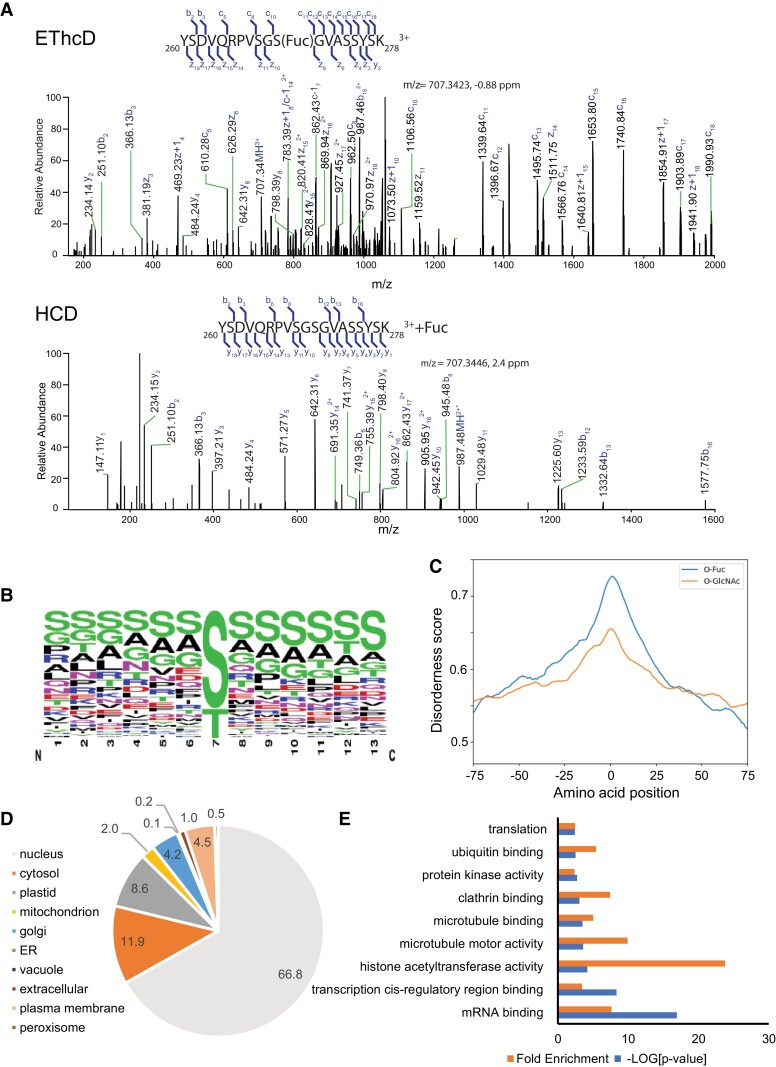
SPY mediates *O*-fucosylation of hundreds of nucleocytoplasmic proteins. A) EThcD and HCD spectra of the peptide spanning amino acid 260–278 of ECT2 with fucosylation modification. EThcD pinpoints the site of *O*-fucosylation on Ser270 of ECT2 based on the mass shift between c10 and c11 ions. HCD identifies the peptide with high confidence, but a neutral loss in the fragment ions prevents the assignment of *O*-fucosylation site. B) The WebLogo shows sequence preference flanking the *O*-fucosylation sites identified in this study. C) Disorderness scores plotted against relative amino acid positions to *O*-fucosylation sites and *O*-GlcNAcylation sites. D) Pie chart shows the experimental and predicted subcellular localization of *O*-fucosylated proteins, analyzed with the subcellular location database for Arabidopsis proteins (SUBA4). Numbers show the percentile of proteins in the indicated subcellular localizations. E) GO analysis of the molecular functions of the *O*-fucosylated proteins.

To identify more *O*-fucosylated proteins, we applied the same AAL-chromatography-MS pipeline to WT without isotope labeling. We analyzed floral tissues (Xu et al. 2017) and seedling tissues (Supplemental Fig. S1). Tissues were harvested from 9 independent biological experiments and 34 mass spectrometry runs were performed using higher-energy collision dissociation (HCD) and electron-transfer/higher-energy collision dissociation (EThcD) analyses (Supplemental Fig. S1). Similar to *O*-GlcNAc (Xu et al. 2017), the *O*-fucose moiety is extremely labile and often dissociates from the precursor during the internal vibronic energy randomization. During HCD fragmentation, the labile bond between the *O*-fucose moiety and the peptide backbone is fragmented before backbone fragmentation; thus, the resulting b and y fragment ions often do not contain *O*-fucose. This neutral loss prevents the use of mass shifts in the peptide sequence ions to establish the site(s) of *O*-fucosylation modification. As such, HCD data often provide a confident assignment of *O*-fucose to a particular peptide but cannot pinpoint the exact site of modification in the sequence (Fig. 3A). In contrast, EThcD (Yu et al. 2017) produces mostly *c*/*z* backbone fragment ions that retain the *O*-fucose moiety, and in many cases allows the assignment of the mass spectrum to a particular peptide sequence and unambiguous site localization of the modification (Fig. 3A).

Our MS analyses of AAL-enriched peptides identified 1750 distinct *O*-fucosylated peptides. These *O*-fucosylated peptides correspond to 943 different peptide sequences from 467 proteins. The data support at least 1072 *O*-fucosylation sites, of which 345 were determined with greater than 95% confidence. A list of these modified peptides and sites of the modification are provided in Supplemental Dataset S5. Sequence analysis of unambiguously assigned *O*-fucosylation sites showed no consensus sequence other than a slight preference for serine in the flanking region (Fig. 3B). The *O*-fucosylation sites tend to be in intrinsically disordered regions; a similar trend was found for previously identified *O*-GlcNAcylated sites (Xu et al. 2017; Fig. 3C). Subcellular localization analysis of the *O*-fucosylated proteins using SUBA4.0 (Hooper et al. 2017) showed a significant enrichment of nuclear proteins (66.8%; *P* = 2.2e-16, fold enrichment = 2.5) (Fig. 3D; Supplemental Dataset S6). In addition, 11.9% and 8.6% of the *O*-fucosylated proteins were predicted to localize in cytosol and plastid, respectively (Fig. 3D; Supplemental Dataset S6). Gene ontology (GO) analysis of protein molecular function supported regulatory roles for *O*-fucosylated proteins. Based on their molecular function annotations from The Arabidopsis Information Resource (TAIR), the *O*-fucosylated proteins showed significant enrichment of key molecular functions including histone modification, transcription, RNA-processing, translation, protein degradation, protein phosphorylation, cytoskeleton, and vesicle trafficking (Fig. 3E; Supplemental Dataset S7).

### Overlap of the *O*-fucosylome with the *O*-GlcNAcome and targets of TOR signaling reveals the integration of nutrient-sensing pathways

**Figure 4.**
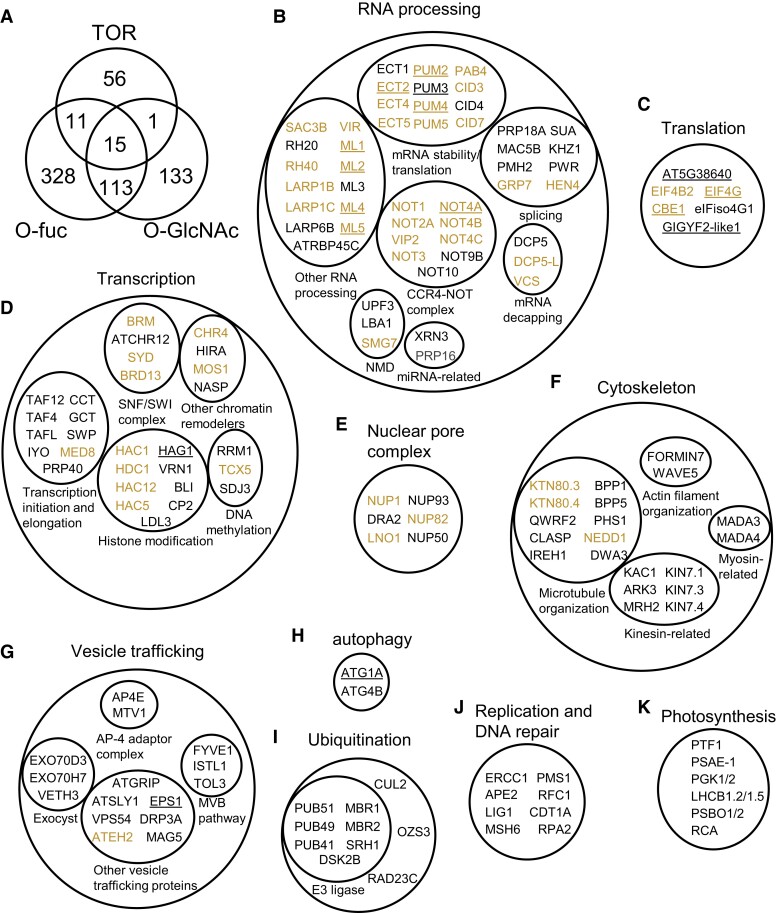
*O*-Fucosylated proteins overlap with *O*-GlcNAcylated proteins and TOR targets in key cellular processes. A) Venn diagram shows overlaps among *O*-fucosylated proteins, *O*-GlcNAcylated proteins, and TOR targets. B–K) Groups of *O*-fucosylated proteins that function in RNA-processing (B), translation (C), transcription (D), nuclear pore complex (E), cytoskeleton (F), vesicle trafficking (G), autophagy (H), ubiquitination (I), replication and DNA repair (J), and photosynthesis (K). *O*-fucosylated proteins that are also *O*-GlcNAc-modified and TOR targets are highlighted by yellow color and underline, respectively.

Previous studies indicated a complex relationship between SPY and SEC, including redundant functions in embryo viability, antagonistic regulation of GA signaling, and unique functions of SPY in various developmental processes (Sun 2021). To understand the relationship between *O*-fucosylation and *O*-GlcNAcylation at the molecular and network levels, we compared the *O*-fucosylome with the *O*-GlcNAcome (Xu et al. 2017). Of the 262 *O*-GlcNAcylated proteins, 128 were also detected as *O*-fucosylated (Fig. 4A). The TOR kinase is another important nutrient sensor. Of the 83 TOR-targeted phosphoproteins (Van Leene et al. 2019), 26 were detected as *O*-fucosylated, 16 were detected as *O*-GlcNAcylated, and 15 were modified by both (Fig. 4A; Supplemental Dataset S8). These results identified groups of proteins that are common targets of the SPY, SEC, and TOR pathways, of two of the three pathways, or potential targets unique to each pathway (Fig. 4A; Supplemental Dataset S8).

Based on known or predicted cellular functions of these groups of proteins, the common targets of SPY, SEC, and TOR are mostly involved in RNA processing and translation (EIF4G and CONSERVED BINDING OF EIF4E1/CBE1) (Fig. 4, B and C). Additionally, the targets shared by SPY and SEC are mostly involved in transcription, nuclear pore complex, and microtubule organization (Fig. 4, D–F). Common targets shared by SPY and TOR include autophagy factor ATG1A and vesicle trafficking protein EPS1 (Fig. 4, G and H). SPY appears to play prominent roles in certain vesicle trafficking processes and has unique roles in DNA replication, DNA repair, protein degradation, and photosynthesis (Fig. 4, I–K).

### Many *O*-fucosylated proteins play important roles in SPY-regulated biological processes

**Figure 5.**
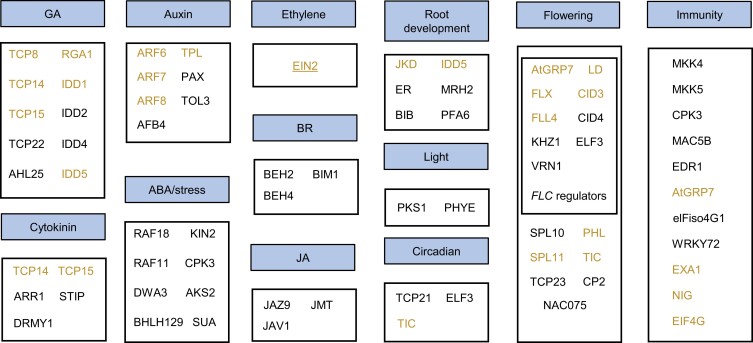
*O*-Fucosylated proteins function in diverse biological processes. Representative groups of *O*-fucosylated proteins with important functions in biological processes. Yellow text and underline mark proteins that are also *O*-GlcNAc-modified and TOR targets, respectively.

Genetic studies have shown that SPY functions in various developmental and physiological processes. Many *O*-fucosylated proteins are known to play important roles in these SPY-regulated processes and are therefore likely downstream mediators of SPY regulation (Fig. 5). The *spy* mutants display slim and dwarf phenotypes and severe growth defects. The *O*-fucosylome data suggests that one major mechanism by which SPY regulates plant growth is through modulation of phytohormone signaling, as components of many phytohormone signaling pathways are modified by *O*-fucose (Fig. 5). SPY *O*-fucosylates and activates DELLA proteins to suppress GA signaling (Zentella et al. 2017). Specifically, SPY was shown to *O*-fucosylate the LSN peptide of RGA (Zentella et al. 2017). The same LSN peptide was also identified as *O*-fucosylated in our *O*-fucose profiling experiment (Supplemental Dataset S5). In addition to RGA, we also detected *O*-fucosylation of many DELLA-interacting proteins. These include several components of the switch defective/sucrose non-fermentable (SWI/SNF) class of ATP-dependent chromatin remodeling complexes (Fig. 4D). Components of the SWI/SNF complex have been reported to interact with SPY and DELLA and play roles in GA responses (Archacki et al. 2013; Sarnowska et al. 2013). DELLA-interacting proteins also include components of other phytohormone signaling pathways (Hu and Ma 2006; Matsushita et al. 2007; Daviere et al. 2014; Fukazawa et al. 2014; Kumar et al. 2019; Fig. 5). These include four members of the INDETERMINATE DOMAIN transcription factors (IDD1, IDD2, IDD4, and IDD5) (Fukazawa et al. 2014; Kumar et al. 2019), and four members of class I TCP transcription factors (TCP8, TCP14, TCP15, and TCP22) involved in GA and cytokinin responses (Daviere et al. 2014; Fukazawa et al. 2014; Kumar et al. 2019), and two members of the BRASSINAZOLE RESISTANT 1 (BZR1) family transcription factors (BES1/BZR1 HOMOLOG 2 (BEH2) and BES1/BZR1 HOMOLOG 4 (BEH4)) involved in brassinosteroid (BR) signaling (Bai et al. 2012), and three auxin response factors (ARF6, ARF7, ARF8) (Oh et al. 2014) (Fig. 5). Additional components other than the DELLA-interacting factors were *O*-fucosylated in the cytokinin, auxin, and BR pathways. Furthermore, we detected *O*-fucosylation of the key component of the ethylene pathway (EIN2) (Alonso et al. 1999), and many proteins involved in the ABA and jasmonic acid (JA) signaling pathways (Fig. 5).

SPY is likely to interact with SEC in regulating phytohormone responses. *O*-fucosylation and *O*-GlcNAcylation of DELLAs have been shown to have opposite effects on their interaction with partner transcription factors such as BZR1 (Zentella et al. 2016, 2017). Both *O*-fucose and *O*-GlcNAc modifications were detected for several TCPs, IDDs, ARFs, and EIN2, suggesting that the two *O*-glycosylation pathways interact not only in GA responses, but also in cytokinin, auxin, and ethylene responses. Interestingly, EIN2 is also a target of TOR signaling (Fu et al. 2021). These results indicate that SPY/*O*-fucosylation modulates all phytohormone responses, in various combinations with the *O*-GlcNAc and TOR pathways.

Phenotypes of the *spy* mutants suggest roles for SPY in light responses, circadian rhythm (Tseng et al. 2004; Wang et al. 2020), root development (Cui et al. 2014; Mutanwad et al. 2020), flowering (Jacobsen and Olszewski 1993), and immunity (Zhang et al. 2019; Sun 2021). Consistent with the genetic evidence, the *O*-fucosylated proteome includes light signaling components such as PHYTOCHROME E (PHYE) (Clack et al. 1994) and phytochrome kinase substrate1 (PKS1) (Fankhauser et al. 1999), proteins involved in the circadian clock such as TCP21 (Pruneda-Paz et al. 2009), TIME FOR COFFEE (TIC) (Hall et al. 2003) and EARLY FLOWERING 3 (ELF3) (Hicks et al. 2001), and many proteins involved in root development, flowering, and immunity (Fig. 5). These results show that SPY/*O*-fucosylation, acting in various combinations with the *O*-GlcNAc and TOR pathways, modulates key cellular activities as well as phytohormone signaling, development, and responses to environmental signals.

## Discussion

As a central nutrient signaling mechanism, *O*-glycosylation of nucleocytoplasmic proteins has been extensively studied in metazoans but has attracted little attention in the field of plant biology. Genetic evidence indicates an essential function of *O*-glycosylation in both kingdoms, as the *ogt* mutants in mammals and the *spy sec* double mutant in Arabidopsis are lethal (O'Donnell et al. 2004; Hartweck et al. 2006). Furthermore, genetic and biochemical studies in *Arabidopsis* have suggested complex interactions between SPY and SEC in a wide range of developmental and physiological processes (Hartweck et al. 2002; Ong et al. 2018). Our study provides genetic evidence for the sugar-signaling function of SPY and a proteomic dataset of *O*-fucosylated proteins in plants, thereby revealing a sugar-signaling network that involves SPY-mediated *O*-fucosylation of hundreds of cellular targets regulating plant growth and development. Our dataset also reveals overlap and potential nodes of crosstalk between the SPY/*O*-fucosylation, SEC/*O*-GlcNAcylation, and TOR signaling pathways.

Studies in animals have shown that the level of *O*-GlcNAc modification of cellular proteins fluctuates with nutrient availability and metabolic status. Whether SPY and SEC activities depend on metabolic status has not been examined experimentally in plants. By growing plants in the dark to deplete endogenous sugars, we showed that exogenous sugar supports seedling growth in a SPY-dependent manner, demonstrating the sugar-sensing function of SPY-mediated *O*-fucosylation. Our results support the notion that sugar availability determines the cellular concentration of GDP-fucose, the donor substrate of SPY, and hence the level of SPY-mediated *O*-fucosylation of nucleocytoplasmic proteins, which mediate sugar regulation of physiological and developmental responses such as growth promotion under sugar-replete conditions and growth arrest or stress responses under sugar-deficient conditions.

We showed that AAL-chromatography specifically enriches *O*-fucosylated peptides. It has been reported that AAL specifically binds to *O*-fucose (Bandini et al. 2016). We developed and optimized an AAL-chromatography procedure to enrich for *O*-fucosylated peptides from *Arabidopsis* samples. Using high-resolution/high-accuracy mass spectrometry, combined with two fragmentation modes and/or isotope labeling, we have identified large-scale *O*-fucosylated peptides with high confidence. The observation that all the quantified *O*-fucosylated peptides were either undetectable or detected at a reduced level in the *spy* mutants further confirms the mass spectrometry identification.

Our proteomic data and the phylogeny of OGTs consistently support that SPY is the only POFUT that catalyzes terminal *O*-fucose modifications in *Arabidopsis*, although the possibility of another POFUT cannot be ruled out. SPY and SEC represent two evolutionarily conserved branches of the OGT family. A single SEC-like OGT catalyzes all *O*-GlcNAc modifications in animals, whereas in Arabidopsis, SEC and SPY, without other homologs (Olszewski et al. 2010; Sun 2021), are likely solely responsible for *O*-GlcNAc and *O*-fucose modifications, respectively. The decreased levels of all the 211 quantified *O*-fucosylated peptides in *spy* provide proteomic evidence supporting that SPY is the only POFUT that catalyzes terminal *O*-fucose modifications in *Arabidopsis.* Detection of some *O*-fucosylated peptides in the *spy* mutants is likely due to partial loss of SPY function in the *spy-23* and *spy-4* alleles, which contain T-DNA insertions around the translation start site and may express a low level of full-length or truncated SPY proteins with catalytic activity. In the absence of evidence for another SPY homolog or isozyme, the proteins that showed a decreased level of *O*-fucosylation in *spy* can be confidently considered as direct SPY substrates, and those *O*-fucosyl peptides un-quantified in our study are most likely also SPY substrates. Our study suggests that the Golgi-localized homologs of mammalian protein *O*-fucosyltransferases (Smith et al. 2018) may not catalyze terminal *O*-fucose modification which is bound by AAL.

Our *O*-fucosylome data includes multiple previously reported SPY substrates and interactors. These include RGA, TCP14, TCP15, and components of the SWI/SNF complex (Sarnowska et al. 2013; Steiner et al. 2016; Zentella et al. 2017). However, we did not detect *O*-fucosylation of PRR5 and AtACINUS (Wang et al. 2020; Bi et al. 2021), suggesting that we achieved only partial coverage of the *O*-fucosylome. On the other hand, the detection of *O*-fucosylation of multiple members of the same protein family (such as ARFs and ECTs, Figs. 4B and 5) suggests the high coverage of our proteomic dataset.

Many *O*-fucosylated proteins have known functions that are consistent with the genetic functions of SPY, and are therefore likely functional targets of SPY. For example, the large overlap between the *O*-fucosylated and *O*-GlcNAcylated proteomes is consistent with the embryo-lethal phenotype of the *spy sec* double mutant (Hartweck et al. 2002). In particular, the overlap represents essential functions such as transcription, RNA processing, translation, as well as auxin and cytokinin signaling which are essential for growth and embryogenesis. Considering that loss of TOR is also lethal, it is interesting to note that the three nutrient signaling pathways overlap in post-transcriptional and translational regulation as well as auxin and ethylene signaling (Deng et al. 2016). The crosstalk among *O*-fucosylation, *O*-GlcNAcylation, and TOR-dependent phosphorylation on these shared target proteins is likely part of the central control system for cell growth.

Energy and nutrient homeostasis involves multiple signaling pathways including *O*-glycosylation and target of rapamycin (TOR) (Sabatini 2017). Exogenous sugar is required for growth after shifting seedlings from light to constant darkness, and such growth-promoting effect of sugar requires both TOR (Zhang et al. 2016) and SPY (Fig. 1). A proteomic study in *Arabidopsis* identified 83 TOR-regulated phosphoproteins (Van Leene et al. 2019). About 31% (26 of 83) of TOR-regulated phosphoproteins are *O*-fucosylated and 19% (16 of 83) are *O*-GlcNAcylated (Fig. 4A). Fifteen TOR-regulated phosphoproteins are both *O*-fucosylated and *O*-GlcNAcylated (Fig. 4A; Supplemental Dataset S8), including EIN2, a key component of the ethylene signaling pathway recently reported to be a substrate of TOR (Fu et al. 2021) and to interact with SPY in tomato (*Solanum lycopersicum*) (Xu et al. 2022). Almost half of the *O*-GlcNAcylated proteins (128 of 262) are *O*-fucosylated. This supports extensive functional overlap between *O*-fucosylation and *O*-GlcNAcylation and also suggests a tight connection between the *O*-glycosylation-mediated and TOR-mediated nutrient signaling pathways. Out of the 26 substrates common to TOR and SPY, nine are RNA-binding proteins and eight out of these nine are also SEC substrates, four are translation regulators, and three out of these four are also SEC substrates (Supplemental Dataset S8). This observation highlights the importance of RNA processing and translation regulation in mediating cellular responses to diverse nutrient signals. The SPY/SEC/TOR co-regulated RNA-binding proteins are mostly from the MEI2-like family and the Pumilio family (Supplemental Dataset S8). In yeast, the *mei2* gene is a master regulator of meiosis and the five *Arabidopsis* MEI2-LIKE (AML) proteins appear to play a similar role in a redundant manner (Kaur et al. 2006). *AML1-5* is highly expressed in the shoot apical meristem, young buds, and reproductive organ primordia (Kaur et al. 2006). Compromising AMLs leads to sterility, developmental arrest caused by defects in meristem activity, and various meiotic chromosome organization defects (Kaur et al. 2006). Similarly, the *Arabidopsis* PUMILIO proteins (APUMs) are required for stem cell maintenance and differentiation, as well as active cell divisions (Abbasi et al. 2011). SPY, SEC, and TOR also co-regulate translation elongation factors and related regulators. Several studies showed that translation elongation factors play a role in cell division and growth (Feng et al. 2007; Zhou et al. 2014; Wang et al. 2016). These genetic and proteomic studies together suggest that the RNA processing and translation factors co-regulated by SPY/SEC/TOR are important regulatory hubs for nutrient-dependent growth and development.

The crosstalk among *O*-fucose, *O*-GlcNAc, and TOR signaling is likely complex and variable for different target proteins. The lethal phenotype of the *spy sec* double mutant suggests that *O*-fucosylation and *O*-GlcNAcylation have similar and additive effects on certain target proteins in contrast to their antagonistic effects on the DELLA proteins and GA responses. Interestingly, SPY is *O*-GlcNAcylated and *O*-fucosylated, suggesting direct crosstalk in addition to the co-regulation of shared targets. Crosstalk between *O*-GlcNAcylation and phosphorylation of the same Ser/Thr residue or nearby residues, as well as complex interactions between the *O*-GlcNAc and TOR pathways, have been observed in metazoans (Hart et al. 2011; Park et al. 2014; Sodi et al. 2015; Very et al. 2018). Our proteomic datasets support many hypotheses and provide molecular targets that can be tested in the future to advance our understanding of the interactions among the nutrient signaling pathways.

The *O*-fucosylome also identified proteins that potentially mediate SPY regulation of phytohormone responses, circadian rhythm, floral transition, light signaling and responses, ABA and stress responses, immunity, root development, and photosynthesis (Fig. 5). The *spy* mutant was initially identified as a GA response mutant and later shown to be hyposensitive to cytokinin (Jacobsen and Olszewski 1993; Greenboim-Wainberg et al. 2005). While SPY's functions in GA and cytokinin responses, through DELLA and TCP factors, respectively, have been studied at the molecular level (Steiner et al. 2012; Zentella et al. 2017), our data reveal a broad integration of SPY/*O*-fucosylation with phytohormone signaling pathways. We found that SPY *O*-fucosylates not only DELLAs but also many proteins that interact with DELLAs, including members of the IDD, ARF, TCP, and BZR1/BES1 families and the SWI/SNF chromatin remodeling complex. How *O*-fucosylation and *O*-GlcNAcylation affect these DELLA-interacting factors and their interaction with DELLAs are interesting questions for future study.

The *spy* mutant shows reduced sensitivity to cytokinin in terms of root growth inhibition, anthocyanin accumulation, leaf margin serration, suppression of inflorescence elongation, and trichome development (Olszewski et al. 2010). Some of these phenotypes were attributed to the SPY-dependent stabilization of TCP14 and TCP15 (Steiner et al. 2012, 2016). The *tcp14 tcp15* double mutant shows a reduced sensitivity to cytokinin-induced leaf margin serration and trichome development, but not suppression of inflorescence elongation (Steiner et al. 2012). Our *O*-fucosylome shows that both TCP14 and TCP15 are *O*-fucosylated by SPY (Fig. 5). In addition, ARR1, a type-B response regulator that mediates cytokinin-dependent transcriptional activation (Hwang et al. 2012), is *O*-fucosylated by SPY. ARR1 could explain the cytokinin-insensitive phenotype of *spy* that appears to be independent of TCP14 and TCP15.

In addition, *O*-fucosylation was detected on components of the BR, auxin, ABA, ethylene, and JA pathways. Many of these components of phytohormone signaling pathways are also modified by *O*-GlcNAc (Fig. 5). These observations indicate that SPY and SEC co-regulate GA, cytokinin, auxin, and ethylene responses, whereas SPY may target unique components in the BR, ABA, and JA pathways.

The *spy* mutants show early flowering phenotypes (Jacobsen and Olszewski 1993; Silverstone et al. 2007), and the *O*-fucosylome includes at least 16 proteins that are known to be involved in various flowering regulation pathways. Eight of these proteins are also *O*-GlcNAcylated (Fig. 5), consistent with the weaker early flowering phenotype of *sec*. Among these eight flowering regulators, GLYCINE RICH PROTEIN 7 (AtGRP7) promotes flowering in *Arabidopsis*, and its wheat homolog TaGRP2 is involved in flowering promotion by vernalization (Streitner et al. 2008; Xiao et al. 2014). Interestingly, vernalization induces *O*-GlcNAcylation of TaGRP2 which contributes to the winter-dependent flowering (Xiao et al. 2014). These observations suggest that SPY may regulate flowering through multiple pathways including the vernalization pathway.

The *spy* mutants have pale leaves, suggesting a defect in chloroplast development and photosynthesis. Intriguingly, the *O*-fucosylome includes 40 chloroplast proteins including 39 encoded by nuclear genes. Interestingly, a recent study showed that SPY mediates *O*-fucosylation of the chloroplast-localized chaperonin CPN20 to reduce its accumulation in the chloroplast (Liang et al. 2021); however, CPN20 was not among the *O*-fucosylated proteins we detected. SPY was previously reported to localize to the nucleus and cytosol (Swain et al. 2002). The proteins may be *O*-fucosylated before being imported into the chloroplast. It is also possible that a fraction of SPY localizes to the chloroplast. In animals, *OGT* is alternatively spliced to produce different forms that localize to the cytosol, nucleus, and mitochondria (Lazarus et al. 2006; Sacoman et al. 2017). Gene annotation in TAIR shows that *SPY* and *SEC* may also be alternatively spliced in *Arabidopsis*, but the localization of these isoforms has not been studied. Interestingly, no *O*-GlcNAcylation was detected on chloroplast proteins (Xu et al. 2017). A unique function of SPY in modifying and regulating chloroplast proteins would be consistent with its evolutionary prominence in photoautotrophic organisms (Olszewski et al. 2010).

In summary, our work uncovers the physiological function of SPY in sugar-dependent growth, the molecular functions of SPY at the proteomic scale, and the cellular targets of *O*-fucosylation. Our findings will pave the way for new discoveries of protein *O*-fucosylation and cross-talks with other pathways and enable functional study of *O*-fucosylation of hundreds of proteins by the plant community.

## Materials and methods

### Plant materials and growth conditions

All the *Arabidopsis thaliana* plants used in this study were in the Col-0 ecotype background. The plants were grown in greenhouses with a 16-h light/8-h dark cycle (about 150 μmol m^−2^ s^−1^ natural light supplemented with white LED light) at 22–24 °C for general growth and seed harvesting. For seedlings grown on the medium in Petri dishes, the sterilized seeds were grown on half-strength Murashige and Skoog (½-MS) medium and supplemented with 0.8% (w/v) phytoagar. Plates were placed in a growth chamber under constant light (about 82 μmol m^−2^ s^−1^ fluorescent bulbs) at 22 °C. T-DNA insertional mutants *spy-23* (WiscDsLox241C03) (Woody et al. 2007), *spy-4*, *sec-2*, and *sec-5* were previously described (Bi et al. 2021).

### Sugar-dependent growth experiment

WT, *spy* and *sec* seeds were sterilized and placed at 4 °C for 2 d. WT and *sec* seeds were transferred onto ½ MS supplemented with 1% (w/v) sucrose or mannitol and placed in a growth chamber under constant light at 22 °C. About 12 h later, the *spy* seeds were transferred onto the same plates to ensure a similar time of germination as the WT and *sec* seeds. Seedlings were grown for 4 d in the growth chamber and then transferred to dark for 8 d. Hypocotyl lengths were measured from scanned images using the ImageJ software, and true leaf development was scored. The results were analyzed by two-way ANOVA in R (length ∼ genotype*sugar; percentage ∼ genotype*sugar). Outputs are provided in Supplemental Dataset S9.

### Bioinformatic analysis

Subcellular localization analysis was performed with SUBA4.0 (https://suba.live/) with default settings (Hooper et al. 2017). The consensus subcellular localization was used.

GO analysis was performed with the **P**rotein **AN**alysis **TH**rough **E**volutionary **R**elationships (PANTHER) Classification System (Mi et al. 2021), using the PANTHER GO-Slim Molecular Function and the PANTHER GO-Slim Biological Process options. Analysis type was set to PANTHER Overrepresentation Test. Repetitive GO terms were removed. The nuclear protein enrichment was calculated with the binom.test function in R.

Disorderness calculation was performed with Protein disorder prediction server (PrDOS) (https://prdos.hgc.jp) (Ishida and Kinoshita 2007).

### Peptide preparation for AAL-enrichment

For quantitative analysis using metabolic stable isotope labeling mass spectrometry (SIL-MS), the Col and *spy* seedlings were grown on ^14^N (½-MS nutrient without nitrogen (PhytoTechnology Laboratories), ^14^NH_4_^14^NO_3_ [0.5 g/L, Sigma], K^14^NO_3_ [0.5 g/L, Sigma], pH 5.7) or ^15^N media (½-MS nutrient without nitrogen, ^15^NH_4_^15^NO_3_ [0.5 g/L, Cambridge Isotope Laboratory], K^15^NO_3_ [0.5 g/L, Cambridge Isotope Laboratory], pH 5.7) for 14 d under constant light at 22 °C. For the SIL-MS quantification experiments comparing WT and *spy-4*, equal amounts of plant tissue powder of the ^14^N and ^15^N samples were mixed before protein extraction, whereas the samples of WT and *spy-23* were mixed after protein extraction.

Flowers were harvested from 5-week-old Col plants grown in greenhouses with a 16-h light/8-h dark cycle at 22–24 °C. The tissues were then ground in liquid nitrogen.

Three volumes (6 ml) of buffer Y (0.1 M Tris⋅HCl, pH 8.0; 2% (w/v) SDS; 20 mM EGTA; 20 mM EDTA; 1.2% (v/v) Triton X-100; 50 mM NaF; 2 × protease inhibitor (Roche); and 40 µM PUGNAc inhibitor (Sigma)) were added to 2 g tissue powder in a 50 ml tube. The samples were vortexed for 1 min and then heated for 10 min at 60 °C. The samples were centrifuged at 20,000 × *g* for 20 min at room temperature (RT) and the supernatant was each transferred to a new 50 ml tube. Equal volume (∼6 ml) of ice-cold phenol (Tris buffered, pH 7.5–7.9) was added and samples were vortexed for 1 min. Samples were centrifuged at 20,000 × *g* at 4 °C for 15 min to separate phenol and aqueous phases. The upper aqueous phase was removed without disturbing the interface. The phenol phase was re-extracted twice with ice cold buffer *Z* (50 mM Tris-HCl, pH 8.0, stored at 4 °C). Five-volume of cold 0.1 M ammonium acetate in methanol was added to the samples and then the samples were incubated at −80 °C overnight. Tubes were centrifuged at 20,000 × *g* for 20 min at 4 °C and supernatant was removed. The pellets were washed with 10 ml cold 0.1 M ammonium acetate in methanol twice and 10 ml cold methanol twice. After removing the trace methanol with pipettes, 1 ml resuspension buffer (6 M Guanidine-HCl, in 25 mM NH_4_HCO_3_, pH 8.0) was added to resuspend the pellets. The samples were each transferred to a new 1.5 ml tube and sonicated at 10% duty cycle for 10 s (1 s ON/OFF) three times (Branson Digital Sonifier 250). *Tris*(2-carboxyethyl)phosphine hydrochloride (0.5 M TCEP, Sigma) was added to the samples to a final concentration of 2 mM and the samples were incubated for 60 min at 56 °C. Iodoacetamide (Sigma, 0.5 M) was added to the samples to a final concentration of 10 mM and the samples were incubated for 60 min at RT in the dark. The samples were diluted with 25 mM NH_4_HCO_3_ to make a final guanidine-HCl concentration of 1.5 M. Protein concentrations were measured by Bio-Rad protein Assay (Bradford). For the ^14^N/^15^N quantification experiments comparing WT and *spy-23*, an equal amount of protein from the ^14^N and ^15^N-labeled samples were mixed. Modified trypsin (Trypsin, TPCK Treated) was added (1: 50 w/w) and the samples were incubated at 37 °C overnight. Modified trypsin was added once again (1:50 w/w) and incubated for an additional 6 h. Next, the protease activity was quenched by acidification of the reaction mixture with formic acid to a final concentration of 1% formic acid. The samples were centrifuged at 20,000 × *g* for 10 min to remove insoluble material. The supernatant was desalted using Sep-PAK C18 cartridges following manufacturer's instructions (Waters). The peptide samples were dried up using SpeedVac (Thermo) and stored at −80 °C before use.

### *O*-fucosylated peptide enrichment with AAL-agarose chromatography

A chromatography column (Tricorn 5/50 Column, Cytiva) was packed with 0.7 ml AAL-agarose (Vector laboratories) following the manufacturer's instructions. Dried peptide samples were resuspended in 105 µl buffer A (PBS + 5% acetonitrile) and 100 µl was loaded. Chromatography was performed with AKTA purifier (GE Healthcare) at a flow rate of 100 µl/min. Buffer B consisted of buffer A with 10 mM L-fucose (Cayman). After washing with seven volumes (4.9 ml) of buffer A, *O*-fucosylated peptides were eluted using buffer B. The eluted fractions were collected and combined for desalting using Sep-PAK C18 cartridges (Waters). The peptide samples were dried using SpeedVac (Thermo) and stored at −80 °C before mass spectrometric analysis.

### MS analysis

For higher-energy collision dissociation (HCD experiments), peptides were analyzed by liquid chromatography-tandem mass spectrometry (LC-MS) on an Easy LC 1200 UPLC liquid chromatography system (Thermo Fisher) connected to a Q-Exactive HF hybrid quadrupole-Orbitrap mass spectrometer (Thermo Fisher). Peptides were separated using analytical Easy-Spray C18 columns (75 μm × 150 mm) (Thermo, ES803). The flow rate was 300 nl/min, and a 120 min gradient was used. Peptides were eluted by a gradient from 3% to 28% solvent B (80% (v/v) acetonitrile/0.1% (v/v) formic acid) over 100 min and from 28% to 44% solvent B over 20 min, followed by a short wash at 90% solvent B. Precursor scan was from mass-to-charge ratio (*m*/*z*) 375 to 1,600 (resolution 120,000; AGC 3.0e6) and the top 20 most intense multiply charged precursors were selected for fragmentation. Peptides were fragmented with HCD with normalized collision energy (NCE) 27.

HCD/EThcD data were acquired on an Orbitrap Eclipse (Thermo Scientific, San Jose, CA, USA) equipped with an Easy LC 1200 UPLC liquid chromatography system (Thermo Fisher). Peptides were fractionated on an analytical Easy-Spray C18 column (75 μm × 150 mm) (Thermo, ES803) using the same gradient as the HCD experiment. Precursor ions were scanned with either two consecutive HCD and EThcD or EThcD only. For both types of scans, the precursor ions were scanned from 375 to 1,600 *m*/*z* (resolution 120,000; AGC 4.0e5) and the charge state 2^+^ to 6^+^ were filtered in the quadrupole with a selection window of 1.0 *m*/*z* and MIPS Peptide filter enabled. For consecutive HCD and EThcD, HCD was carried out at collision energy of 27% measured in Orbitrap with 60 ms maximum injection time and 1 micro scan (resolution 15,000; AGC 1.0e4). The peptides were then subjected to EThcD fragmentation with maximum injection time of 100 ms, supplemental activation collision energy of 35% measured in the Orbitrap with 3 micro scans (resolution 15,000; AGC 5.0e4). The overall scan cycle was 3 s. For EThcD only scan the precursors were subjected to EThcD fragmentation with 35% supplemental activation collision energy and 200 ms maximum injection time (resolution 15,000, AGC 15.0e4). The number of microscans and scan cycle were the same as with the sequential EThcD.

MS/MS data were converted to peaklist using a script PAVA (peaklist generator that provides centroid MS2 peaklist) (Guan et al. 2011; Shrestha et al. 2022), and data were searched using Protein Prospector against the TAIR database *Arabidopsis thaliana* from December 2010 (https://www.arabidopsis.org/), concatenated with sequence randomized versions of each protein (a total of 35,386 entries). A precursor mass tolerance was set to 5 ppm and MS/MS2 tolerance was set to 20 ppm. Carbamidomethylcysteine was searched as a constant modification. Variable modifications included protein N-terminal acetylation, peptide N-terminal glutamine (Gln) conversion to pyroglutamate, methionine (Met) oxidation, as well as *O*-fucosylation of serine and threonine and single, double, and triple neutral loss of *O*-fucosylation. ^15^N-labeled searches were done the same as mentioned above, considering all 20 amino acids are constantly modified by ^15^N labeling. False discovery rate (FDR) was set to 1% for both proteins and peptides. For quantification, ^15^N labeling efficiency was manually checked. “^15^N labeling” was chosen as a quantitative method using Protein Prospector with automatic adjustment of L:H intensity ratios with labeling efficiency. The cleavage specificity was set to trypsin, allowing two missed cleavages and a maximum of three modifications. False discovery rate was less than 1% at the peptide level according to target:decoy database searching.

Quantification of data from the ^14^N/^15^N reverse labeling experiment was manually checked to correct data points with wrong peak calling. The background signal intensity was set to 1,000 and the expect value cut-off was set to 0.00001. Peptides that showed median WT/*spy* ratios with greater than 100-fold difference in the forward and reverse labeling experiments were removed as inconsistent measurements. The consistently quantified median WT/*spy* ratios of *O*-fucosylated and non-*O*-fucosylated peptides were shown in scatter plots.

The *O*-fucosylated peptide list was filtered sequentially to reduce false positives: (i) From proteins with more than one unique mass peptide; (ii) From proteins with one unique mass peptide but identified by both HCD and EThcD; (iii) From proteins with one unique mass peptide, which had expectation value <= 1.0e-6; (iv) From *O*-GlcNAc-modified proteins and spectrum quality was manually inspected. Peptides that passed these filters were combined into the final list of *O*-fucosylated peptides.

### Accession numbers

The mass spectrometry proteomics data are available via PRIDE, with accession numbers PXD038490 and PXD038491.

SPY (AT3G11540); SEC(AT3G04240)

## Supplemental data

The following materials are available in the online version of this article.

**Supplemental Fig. S1.** Summary of AAL-enrichment and MS experiments.

**Supplemental Dataset S1.** Quantification of peptides in WT vs *spy-4* stable isotope labeling mass spectrometry (SIL-MS) experiments.

**Supplemental Dataset S2.** Median WT/*spy-4* signal ratios of peptides detected and quantified in both isotope-switched replicate experiments.

**Supplemental Dataset S3.** Quantification of peptides in WT vs *spy-23* stable isotope labeling mass spectrometry (SIL-MS) experiments.

**Supplemental Dataset S4.** Median WT/*spy-23* signal ratios of peptides detected and quantified in both isotope-switched replicate experiments.

**Supplemental Dataset S5.** A compiled list of all *O*-fucosylated peptides.

**Supplemental Dataset S6.** Subcellular localization of *O*-fucosylated proteins predicted by SUBA 4.0.

**Supplemental Dataset S7.** GO analysis of *O*-fucosylated proteins with PANTHER.

**Supplemental Dataset S8.** Overlaps among *O*-fucosylated proteins, *O*-GlcNAcylated proteins and TOR-targeted phosphoproteins.

**Supplemental Dataset S9.** Two-way ANOVA analysis of sucrose-dependent growth phenotypes.

## Supplementary Material

koad023_Supplementary_Data
