# Supplementary material for: SPINDLY mediates O-fucosylation of hundreds of proteins and sugar-dependent growth in Arabidopsis
Source: Plant Cell. 2023 Feb 6;35(5):1318–33. doi: 10.1093/plcell/koad023 (PMC10118272; doi:10.1093/plcell/koad023)
Supplement: koad023_Supplementary_Data [file koad023_supplementary_data.zip › Supplemental Figure S1.pdf]

Sample Description			Fragmentation Modes	File Name	L/H ratio (Normalization)	Tissue Types
Sample **1**	Col	$[^{14}N]$	HCD	[Q20200915_02]	2.03	14 days seedlings
	spy-4	$[^{15}N]$	HCD/EThcD	[E20200930_01]		
			EThcD	[E20201009_04, E20201009_05]		
Sample **2**	Col	$[^{15}N]$	HCD	[Q20200918_02]	1.00	14 days seedlings
	spy-4	$[^{14}N]$	HCD/EThcD	[E20200930_03]		
			EThcD	[E20201009_06, E20201009_07]		
Sample **3**	Col	$[^{14}N]$	HCD	[Q20210803_02]	1.40	14 days seedlings
	spy-4	$[^{15}N]$	HCD/EThcD	[E20210805_01, E20210805_02]		
Sample **4**	Col	$[^{15}N]$	HCD	[Q20210803_05]	1.05	14 days seedlings
	spy-4	$[^{14}N]$	HCD/EThcD	[E20210805_03]		
Sample **5**	Col	$[^{14}N]$	HCD	[Q20200915_04]	1.69	14 days seedlings
	spy-23	$[^{15}N]$	HCD/EThcD	[E20200930_07]		
			EThcD	[E20201009_10, E20201009_14]		
Sample **6**	Col	$[^{14}N]$	HCD	[Q20200930_01]	1.44	14 days seedlings
	spy-23	$[^{15}N]$	HCD/EThcD	[E20200930_09]		
			EThcD	[E20201009_12]		
Sample **7**	Col	$[^{15}N]$	HCD	[Q20200930_03]	0.98	14 days seedlings
	spy-23	$[^{14}N]$	HCD/EThcD	[E20200930_11]		
			EThcD	[E20201009_13]		
Sample **8**	Col Flower		HCD	[Q20200918_05]	n.a.	Inflorescence tissues
			HCD/EThcD	[E20200930_05]		
			EThcD	[E20200930_23, E20201009_02, E20201009_03, E20201009_08, E20201009_09, E20201009_11]		
Sample **9**	Col Flower		HCD	[Q20200930_05]	n.a.	Inflorescence tissues
			HCD/EThcD	[E20200930_13]		
			EThcD	[E20200930_24]		

Supplemental Figure S1. Summary of AAL-enrichment and MS experiments (Supports Figure 2). Proteins were extracted from Arabidopsis flowers or seedlings, followed by trypsin digestion and AAL enrichment. To determine if the O-fucosylation is SPY-dependent, seedling samples were metabolically abeled for quantification. As samples were not mixed exactly at a 1:1 ratio, the 14N/15N ratio for each mixture is determined which was used for ratio normalization. The prepared peptides were analyzed with two MS instruments (Q: Orbitrap Q Exactive HF; E: Orbitrap Eclipse) and fragmented with HCD or EThcD, or combined modes.
