# Supplementary material for: SPINDLY mediates O-fucosylation of hundreds of proteins and sugar-dependent growth in Arabidopsis
Source: Plant Cell. 2023 Feb 6;35(5):1318–33. doi: 10.1093/plcell/koad023 (PMC10118272; doi:10.1093/plcell/koad023)
Supplement: koad023_Supplementary_Data [file tpc22.01177.PeerReviewReport.pdf › tpc22.01177.PeerReviewReport.pdf]

# SPY mediates O-fucose modification of hundreds of proteins and sugar-dependent growth in Arabidopsis

Yang Bi, Ruben Shrestha, Zhenzhen Zhang, Chuan-Chih Hsu, Andres V Reyes, Sumudu Karunadasa, Peter R Baker, Jason C Maynard, Yang Liu, Amirmansoor Hakimi, Daniel Lopez-Ferrer, Tahmid Hassan, Robert Chalkley, Shou-Ling Xu, Zhi-Yong Wang

*Corresponding authors: Shou-Ling Xu (xslxu@stanford.edu) and Zhi-Yong Wang (zywang24@stanford.edu).*

**Review timeline:**

| | | |
|---|---|---|
| **TPC2022-RA-00779** | Submission received: | Aug. 14, 2022 |
| | 1st Decision: | Sept. 15, 2022 *manuscript declined* |
| **TPC2022-BR-00970** | Submission received: | Oct. 2, 2022 |
| | 1st Decision: | Nov. 2, 2022 *manuscript declined* |
| **TPC2022-BR-01177** | Submission received: | Dec. 2, 2022 |
| | 1st Decision: | Dec. 12, 2022 *acceptance pending, sent to science editor* |
| | Final acceptance: | Jan 21, 2023 |

**REPORT:** (The report shows the major requests for revision and author responses. Minor comments for revision and miscellaneous correspondence are not included. The original format may not be reflected in this compilation, but the reviewer comments and author responses are not edited, except to correct minor typographical or spelling errors that could be a source of ambiguity.)

## TPC2022-RA-00779 1st Editorial decision – *declined* Sept. 15, 2022

First, I apologize for the length of time that it took to get feedback to you. We aim to be quicker, but your manuscript arrived as a number of editors were traveling or starting the new semester, so handling has been a bit slower these last few weeks.

Thank you for submitting your manuscript entitled "SPY mediates O-fucose modification of hundreds of proteins and sugar-dependent growth in Arabidopsis" to The Plant Cell. Your submission has been evaluated by members of the editorial board, and we regret to inform you that we are not recommending that your manuscript in its current form proceed further in the review process. However, we would like to encourage you to resubmit a revised version of your manuscript to The Plant Cell.

Your submission was assessed at this stage by four editorial board members. The editors judged that the current version of the manuscript is not developed enough at the mechanistic and biological levels as would be expected for a Plant Cell research article. Nevertheless, the identification of fucosylation as a read-out of sugar response, and identification of a large number of proteins that are modified, could potentially be the starting point for further mechanistic investigations. We would reconsider this manuscript as a "Breakthrough Report" around o-fucosylome; this, however, would need reformatting to fit that category.

If you would be willing to rewrite/reformat to fit as a "Breakthrough Report," we will be happy to reconsider the manuscript. We thank you for your interest in and support of The Plant Cell.

## TPC2022-BR-00970 Submission received Oct. 2, 2022

Reviewer comments on previously declined manuscript and **author responses:**

> **Thank you for suggesting reforming our manuscript as a "Breakthrough Report". We have reformatted our manuscript into a "Breakthrough Report" according to the instructions of The Plant Cell. We hope that our manuscript can be published in The Plant Cell soon.**

## TPC2022-BR-00970 1st Editorial decision – *declined* Nov. 2, 2022

Thank you for choosing to send your manuscript entitled "SPY mediates O-fucose modification of hundreds of proteins and sugar-dependent growth in Arabidopsis" for consideration at The Plant Cell. Your submission has been evaluated by members of the editorial board as well as expert reviewers in your field, and we regret to inform you that we are not able to recommend the publication of this manuscript in its current form. We have had input from multiple scientists and we have solicited post-review comments. That said, we would be willing to re-consider a revised manuscript that fully addresses the concerns raised during this review process.

As you will see from their comments, all reviewers judge that your analysis was performed according to the highest technical standards. They suggest, however, several improvements to the data presentation and data organization, and rate that some findings could be better embedded in the paper (sucrose effects), be better and more carefully discussed (direct regulation by SPY) and be more clearly presented (see comments reviewer #1), and some replication of key experiments (Reviewer 2). The reviewers did feel that your work is too descriptive and requested at least some experimental evidence that the proposed modifications have a functional role; the editors discussed this point, and recognizing that the paper is being considered in the context of a Breakthrough Report, we feel that adding functional data is highly recommended, but not essential, as the "BR" papers may be less complete stories.

-------------------------------------------------------------------------- Reviewer comments:

[Reviewer comments shown below along with author responses]

| **TPC2022-RA-01177 Submission received** | **Dec. 2, 2022** |
|---|---|

Reviewer comments on previously declined manuscript and **author responses:**

Reviewer #1:

The authors describe an interesting experiment that led them to the discovery of a reasonably large number of fucosylated proteins it AT. The work appears well executed as one would expect given the experience of the main authors in this context. The list of differentially fucosylated proteins (wt vs SPY knockout) provide a starting point for further work by the authors or other members of the community. That said, I have a few reservations that need to be clarified.

**Response: Thank you for your positive comments on the quality and impact of our work.**

The list of fucosylated proteins is certainly interesting per se. But the work appears to be entirely descriptive. For a journal such as Plant Cell, one would expect to see at least an example for what this differential fucosylation may mean in functional terms.

**Response: Our work uncovers the physiological function of SPY in sugar-dependent growth, the molecular functions of SPY at the proteomic scale, and the cellular targets of O-fucosylation. Several examples for the function of O-fucosylation have been reported, including the DELLA proteins of the GA signaling pathway (Zentella et al., 2017), PRR5 (Wang et al., 2020), ACINUS (Bi et al., 2021) and CPN20 (Liang et al., 2021). Our dataset will enable functional study of O-fucosylation of hundreds of proteins by the plant research community.**

Figure 2: Panels D-F are not really needed and should be moved to the supplement.

**Response: We agree that Fig 2D-2F are not essential. However, we think these examples (together with Fig 2C) of spectra results help readers who are not experts in mass spectrometry to appreciate the quality of the results presented in Fig 2G and 2H.**

Figure 3: the spectra are too busy to be useful as a main figure item. I suggest to show panel B only as this contains the important information and scale it across the entire width of the page to make the spectrum annotations clearer

**Response: we have scaled both panel A and B across the entire width of the figure. We think it is informative to compare HCD with EThcD in the same figure. Breakthrough Reports recommend all figures, if possible, to be in the main figure.**

Panels D and E could be combined into one graph for easier comparing/contrasting the two sugar modifications.

**Response: We have combined D and E into one graph.**

Panel G appears to contain redundant information. Please clean up.

**Response: We thank the reviewer's suggestion and have removed "polyubiquitination-dependent protein binding" category.**

Figure 4 and related text: have the authors checked that these overlaps of fucosylated proteins with e.g. mTOR pathway members are actually statistically significant? gene set enrichment p-values are plagued by over-optimistic statistics.

**Response: Yes, the overlap is statistically significant. We have identified 467 O-fucosylated proteins, which is about 3% of the intracellular proteins encoded in Arabidopsis genome, yet about ½ of O-GlcNAc-modified proteins and ⅓ of mTOR pathway members are O-fucosylated. If we assume all intracellular Arabidopsis proteins could potentially be targets of O-fucose/O-GlcNAc modification and mTOR, the probability of having such a large overlap by chance is $p<2.2e-16$ (binomial test).**

Figure 5: do the authors think it is necessary to show this? It is not immediately clear to this reviewer what the message of the figure is supposed to be.

**Response: This figure shows O-fucosylated proteins that are related to the various *spy* mutant phenotypes (known biological functions of SPY). The messages are that the O-fucosylation of key components of these regulatory pathways is consistent with the known genetic functions of SPY in these biological processes, and thus (1) these target proteins represent important functions of SPY/O-fucosylation, (2) they should be high-priority candidates for future functional and mechanistic studies, (3) researchers who study these proteins or the biological processes should be aware of these O-fucose modifications and the connection to SPY and nutrient signaling.**

The authors should downplay the interpretation that all differentially fucosylated proteins are direct substrates of SPY unless it is clear that there are no other fucosyl-transferases downstream of SPY that may the enzymes responsible for the actual modification. At least for phosphorylation-regulated pathways we know that knocking out one kinase may not mean that all proteins with altered phosphorylation are substrates of that kinase.

**Response: There is no evidence for another nucleocytoplasmic O-fucosyltransferase. Each plant and animal genome encodes many kinases but only one SEC-like OGT and one SPY-like O-fucosyltransferase, with the latter present only in plants and microbes. OGT in animals has been studied extensively and so far, is the only enzyme able to catalyze the O-GlcNAc modification. SPY, but not SEC, is reported to possess O-fucose transferase activity. Thus, existing literature and our result suggest that SPY is the only nucleocytoplasmic O-fucosyl-transferase. The situation is clearly different from kinases.**

**However, we agree to downplay our interpretation as the following:**

**Abstract: All the O-fucosylation events quantified in proteomic analyses are undetectable or dramatically decreased in the *spy* mutants and thus likely catalyzed by SPY.**

**Discussion: Our proteomic data and the phylogeny of OGTs consistently support that SPY is the only POFUT that catalyzes terminal O-fucose modifications in *Arabidopsis, although* the possibility of another POFUT cannot be ruled out. SPY and SEC represent two evolutionarily conserved branches of the OGT family. The single SEC-like OGT catalyzes all O-GlcNAc modifications in animals, whereas in Arabidopsis, SEC and SPY, without other homologs (Olszewski et al., 2010; Sun, 2021), are likely solely responsible for O-GlcNAc and O-fucose modifications, respectively… In the absence of evidence for another SPY homolog or isozyme, the proteins that showed a decreased level of O-fucosylation in *spy* can be confidently considered as direct substrates of SPY, and those O-fucosyl peptides un-quantified in our study are most likely also SPY substrates.**

<u>Reviewer #2</u>:

This paper reports two significant contributions. 467 proteins O-fucosylated by SPY, a protein that impacts many plant physiological processes, were identified using AAL lectin affinity chromatography, a method developed for this study followed by quantitative mass spectrometry. Sucrose is shown to increase O-fucosylation and modulate growth

in a SPY-dependent manner. These contribution are significant because they provide insights into how SPY regulates plant processes through O-fucosylation and connect this regulation to nutrition (sucrose).

The studies to identifying O-fucosylated proteins are solid. The identification methods are validated in several ways which gives high confidence to the identifications.

**Response: thank you for recognizing the significance and quality of our work.**

The evidence for sucrose increasing O-fucosylation is based on a single blot that does not appear to have been replicated and thus is preliminary. In addition to replicating the experiment the signal on the blot should be quantitated and analyzed statistically to provide stronger support for the conclusion.

**Response: We repeated the experiments six times but were unable to reproduce the same clean results. Repeats 2 to 6 used a new AAL reagent and all yielded smear bands that are the same between WT and spy. It seems that the new AAL reagent yields a high level of non-specific signal. We decide to remove this data, not only because we cannot reproduce the result but also because it's a well-established concept that sugar availability affects the level of GDP-fucose and thus SPY-mediated protein O-fucosylation. OGT-catalyzed O-GlcNAc modification is known to be dependent on donor substrate (UDP-GlcNAc) and nutrient level.**

The studies of sucrose, SPY and O-fucosylation identify a new role for SPY and suggest that nutrition regulates the modification abundance but they are not well connected to the rest of the paper. A section should be added to the Discussion bringing these two parts of the paper together.

**Response: We added at the end of 2nd paragraph of Discussion: "Our results support the notion that sugar availability determines the cellular concentration of GDP-fucose, the donor substrate of SPY, and hence the level of SPY-mediated O-fucosylation of nucleocytoplasmic proteins, which mediates sugar regulation of physiological and developmental responses such as growth promotion under sugar-sufficient conditions and growth arrest or stress responses under sugar-starvation conditions." We also added in the 7th paragraph: "Exogenous sugar is required for growth after shifting seedlings from light to constant darkness, and such growth-promoting effect of sugar requires both TOR (Zhang et al., 2016) and SPY (Figure 1)."**

Other comments:

Line 31. Rather than showing an effect of sucrose on per se SPY activity, the experiment shows that there is more modification. This could be due to modulation of SPY activity or an increase in GDP-fucose levels.

**Response: We changed the sentence to : "we demonstrate that SPY mediates sugar-dependent growth."**

Line 60. This study...

**Response: Corrected in the manuscript.**

Line 62. nucleocytoplasmic

**Response: Corrected in the manuscript.**

Figure 1 and line 134. Mannitol is a sugar alcohol so the media is not sugar-free. You should explain how mannitol is being used as a control. It is being used as an osmotic control? Probably not a big concern here but 1% mannitol and sucrose have different osmotic potentials.

**Response: The experiment was done with 1% sucrose and 1% mannitol. We replaced sugar-free with mannitol-containing in this paragraph.**

Line 140-142 and Figure 1E. Are you sure the AAL-binding protein you mark as being absent in spy are absent or just have a reduced mobility in spy. Looking at the blot the pattern of signal suggests this might be happening. That said I can't come up with a good hypothesis how this can happen and the protein remain AAL-detectable. Maybe they react non-specifically. Have you test if fucose blocks the binding to these proteins?

**Response: We removed Figure 1E, as explained above.**

Line 161. What are the proteins that are fucosylated in spy? How confident are you that they are modified?

**Response: The identification by mass spectrometry is highly confident. Further, all these O-fucosylated peptides were reduced in the spy alleles used in this study. Both *spy-23* and *spy-4* contain T-DNA insertions in the 5' end and could produce truncated SPY proteins with a residual activity that accounts for the O-fucosylated peptides in spy mutants. This is likely the case as we have recently identified a spy allele with more severe phenotypes than *spy-4* and *spy-23*.**

Line 320. Is it possible that PRR5 and AtACINUS are not present in the tissues examined in this study?

**Response: PRR5 protein level is clock-regulated, and our plants were grown under continuous light, so it is possible that the PRR5 level is low in our sample. AtACINUS is ubiquitously expressed. Our data has not achieved full coverage of all O-fucosylated proteins, as each of the 34 MS analyses of 9 biological samples detected some new O-fucosylated peptides.**

Line 355. This might be a good place to bring in the sucrose effect shown in Figure 1.

**Response: Do you mean line 335? We added the following discussion: Sugar is required for growth after shifting seedlings from light to constant darkness, and such growth-promoting effect of sugar requires both TOR (Zhang et al., 2016) and SPY (Figure 1).**

Line 411. Are the nine plastid localized proteins all encoded by the nuclear genome? If they are it is possible that they are modified before being imported into the chloroplast.

**Response: All 9 are encoded by the nuclear genome; we have added a sentence to Discussion: It is possible that the proteins are O-fucosylated before being imported into the chloroplast.**

Figure 2D. Are the L/H ratios on the figure correct? By eye they seem wrong.

**Response: The ratios were normalized to the N14:N15 ratio shown in Supplementary Figure 1. We changed to ratios before normalization to avoid confusion.**

It is interesting that SPY is both O-GlcNAc and O-fucose modified.

**Response: Thank you. We added in discussion: Interestingly, SPY itself is O-GlcNAcylated and O-fucosylated, suggesting direct crosstalk in addition to the co-regulation of shared targets.**

Reviewer #3:

In this manuscript, the authors identify hundreds of O-fucosylated proteins as substrates of SPY using lectin affinity chromatography followed by quantitative mass spectrometry. Many O-fucosylated proteins are also modified by O-GlcNac and by phosphorylation of TOR. Genetic analysis revealed that SPY activity is dependent on sugar. The data presented in the manuscript is clear, original and will be interesting for a broad audience of plant biologists. Some of the findings could be further supported by addressing the following concerns:

1. Using the seedlings of wild type and spy-23 or spy-4 with stable isotope labeling mass spectrometry, the authors identified 116 and 170 O-fucosylated proteins, respectively. Using the inflorescence tissues of wild type plants without isotope labeling, 467 O-fucosylated proteins were identified. What is the overlap of O-fucosylated proteins among these three experiments? It seems that the O-fucosylated proteins using spy-23 or spy-4 mutant were more likely the substrates of SPY. Why did the authors not use these proteins for subsequent analysis.

**Response: We added in the Result: "The two datasets overlap by 75 and together show SPY-dependent O-fucosylation of 211 peptides". The 467 O-fucosylated proteins were the total identified in seedlings and floral tissue after 34 MS runs of samples from 9 biological experiments. Because MS analysis doesn't identify all peptides, it is not really meaningful, and it can even be misleading, to present the overlap among the partial coverage from different experiments or tissues. Because isotope labeling increases complexity and reduces data coverage, we performed additional MS analysis without quantitative comparison to spy mutants. However, the observation that 100% of the 211 quantified O-fucosylated peptides are SPY-dependent indicates an extremely high probability for the additional unquantified O-fucosylated peptides to be also SPY-dependent. Such strict SPY dependence is also expected based on animal literature and the phylogeny of SPY and SEC. OGT in animals has been studied extensively and so far, is the only enzyme that is able to catalyze the O-GlcNAc modification. SPY and SEC are the**

**only known OGT homologs in Arabidopsis. SPY, but not SEC, shows O-fucose transferase activity. Thus, both existing literature and our result support that SPY is the only O-fucosyltransferase in Arabidopsis. As the additional unquantified O-fucosylated proteins are most likely also SPY substrates, it is reasonable to use the combined list of 467 O-fucosylated proteins as putative SPY substrates for subsequent analysis.**

2. Genetic analysis showed that SPY is required for sucrose-promoted cell elongation. How sugar regulates SPY activity? In addition, the statistical analysis is required for Fig. 1C.

**Response: We added to the 2nd paragraph of Discussion: "Our results support the notion that sugar availability determines the cellular concentration of GDP-fucose, the donor substrate of SPY, and hence the level of SPY-mediated O-fucosylation of nucleocytoplasmic proteins, which mediates sugar regulation of physiological and developmental responses such as growth promotion under sugar-sufficient conditions and growth arrest or stress responses under sugar-starvation conditions." And We have added statistical analysis in Figure 1C.**

3. The title of Fig. 3 is "SPY mediates O-fucosylation of hundreds of nucleocytoplasmic proteins". However, in the figure 3, there is no any experimental evidence to support that O-fucosylation regulates the subcellular localization of target proteins.

**Response: The title means that "SPY mediates O-fucosylation of hundreds of cytoplasmic and nuclear proteins", to distinguish from the O-fucosylation of secreted proteins. We didn't make any claim on O-fucosylation-mediated regulation of protein localizations.**

4. Similar to Figure 3, the title of figure 4 is not match to the content. Only the Fig. 4A describes the overlap between O-fucosylation, O-GlcNac and targets of TOR. Figure 4B-4K all introduced that the O-fucosylation proteins are involved in different plant biological processes.

**Response: In figure 4B-K, the O-GlcNAc-modified proteins and TOR targets are highlighted by yellow color and underline, respectively.**

5. Hundreds of O-fucosylation proteins were identified by AAL-based chromatography. It will be better to provide some experimental evidence to verify that some target proteins are indeed modified by SPY-mediated O-fucosylation, and this modification regulates the activities of target proteins in plants.

**Response: Regulatory functions of SPY-mediated O-fucosylation have been reported for several proteins (e.g. RGA, PRR5, ACINUS and CPN20). For the RGA protein, our experiment actually identified O-fucosylation of the same peptide that was previously reported. Our O-fucosylome dataset is of high confidence and will enable future functional studies of how O-fucosylation regulates various proteins and biological processes, each of which will be a separate story.**

**TPC2022-RA-01177 1st Editorial decision – *acceptance pending* Dec. 12, 2022**

We are pleased to inform you that your paper entitled "SPY mediates O-fucose modification of hundreds of proteins and sugar-dependent growth in Arabidopsis" has been accepted for publication in The Plant Cell, pending a final editorial review by a science editor.

**Final acceptance from Science Editor Jan. 21, 2023**
